# Antimicrobial activity, ergosterol content and phytochemical screening of *Rorippa Islandica* (Oeder ex Murr.) and *Carrichtera annua* (L.)

**DOI:** 10.1038/s41598-025-18681-9

**Published:** 2025-10-07

**Authors:** Heba Yehia, Marwa A. Ibrahim, Samah A. Donia, Naziha M. Hassanein, Enas Ibrahim

**Affiliations:** 1 Medicinal and Aromatic Plants Department, Desert Research Center, Cairo, Egypt; 2 Microbiology Department, Faculty of Science, Ain Shams University, Cairo, Egypt

**Keywords:** Non-polluting, Phytochemical, Fungal diseases, Antifungal activity, Microbiology, Plant sciences

## Abstract

The purpose of this study was to identify non-polluting medicinal plant alternatives. Two medicinal plants, *Rorippa islandica* and *Carrichtera annua* (family Brassicaceae), collected from the NorthWestern Coastal region (Marsa Matrouh, Egypt), were investigated to determine their bioactive constituents in ethanol crude extracts using GC-MS and HPLC techniques, and to evaluate their in vitro antifungal activity against five pathogenic fungi. GC-MS analysis revealed the presence of 50 and 54 compounds in *R. islandica* and *C. annua*, respectively. The primary compound in *R. islandica* was 13-Docosenamide (20.54%), while in *C. annua* it was 2-Hydroxy-1-(hydroxymethyl) ethyl stearate (9.74%). HPLC identified 19 and 18 phenolic compounds in *R. islandica* and *C. annua*, respectively, with gallic acid as the predominant compound in both plants (3417.72 µg/g and 3733.98 µg/g, respectively). Both plant extracts exhibited promising antifungal activity. For *R. islandica*, the most potent effect at 10 mg/ml was observed with the 70% ethanol successive extract against *Colletotrichum gloeosporioides* (inhibition zone 29 ± 0.3 mm; MIC 7.8 µg/ml; MFC 15.62 µg/ml). For *C. annua*, the most potent effect at 10 mg/ml was from the total extract against *Curvularia lunata* (inhibition zone 35 ± 0.1 mm; MIC 1.97 µg/ml; MFC 3.9 µg/ml). A significant reduction in ergosterol content was observed in the total and successive ethanol (96% and 70%) fractions of both plants, with the highest reduction in *C. lunata* and *C. gloeosporioides* treated with *C. annua* extract (49.93% and 47.7%, respectively), and in *P. glabrum* and *C. gloeosporioides* treated with *R. islandica* extract (47.2% and 42.58%, respectively). Morphological changes induced by the total and ethanol (96% and 70%) extracts of both plants were examined using AFM.

## Introduction

Humans have long relied on biodiversity for various resources. However, conventional agriculture has often utilized chemical pesticides extensively and improperly, leading to significant negative consequences. These practices have resulted in acute and chronic toxicity in humans, widespread environmental contamination, and the development of increased resistance in target pests, ultimately leading to the emergence of more harmful species^1^. Consequently, regulatory bodies are increasingly focused on protecting human, animal, and environmental health from the risks associated with improper pesticide use.

Fungal diseases remain a major threat to crop yields worldwide. While chemical fungicides have been the standard solution, their use poses risks to consumers and the environment, particularly when pre-harvest intervals are not observed^2^. This has driven a global trend toward developing ecologically friendly and safe integrated crop management (ICM) strategies^3^. A key component of this shift is the growing interest in biopesticides—natural pesticides derived from living organisms or natural substances^4^. Plant extracts, in particular, offer a promising, environmentally benign alternative for comprehensive pathogen control^3^.

The antimicrobial action of plant-derived compounds, or phytochemicals, is supported by diverse mechanisms. These compounds can disrupt cellular metabolism (e.g., cinnamaldehyde), rupture microbial membranes (e.g., eugenol and thymol), inhibit biofilm formation (e.g., geraniol and carvacrol), and reduce the production of microbial toxins (e.g., tannin)^5,6^. Additionally, some plant metabolites can decrease the ergosterol content in fungal cell membranes, causing the leakage of vital cellular contents like proteins and nucleic acids^7^.

A significant family of plants with recognized antimicrobial potential is the Brassicaceae (Cruciferae) family. These plants are known for synthesizing and accumulating glucosinolates (GSLs), sulfur-containing compounds that are crucial for plant defense^8^. The hydrolysis of GSLs yields isothiocyanates (ITCs), which have been widely studied for their diverse biological activities, including herbicidal, insecticidal, nematocidal, and, most importantly for this study, antifungal and antibacterial properties^9^.

The present study investigates the antifungal efficacy of plant extracts from *R. islandica* and *C. annua L.*, both members of the Brassicaceae family. The extracts were evaluated against Five significant phytopathogens: *A. cerealis* MT808477 (tomato leaf spot)^10^, *F. solani* OK464437 (fusarium wilt)^11^, *C. lunata* OM432028 (maize leaf spot)^12^, *P. glabrum* Op694171 (pomegranate postharvest rot)^13^, and *C. gloeosporioides* OP177948 (anthracnose)^14^. Despite previous findings suggesting that *Rorippa islandica* and *Carrichtera annua* are rich in bioactive secondary metabolites with promising antimicrobial properties, a comprehensive understanding of their chemical profiles and antifungal efficacy against a wide spectrum of phytopathogenic fungi remains limited. This research aims to assess the potential of these extracts as non-polluting alternatives to chemical fungicides by evaluating their antifungal activity and analyzing their chemical composition.

## Materials and methods

### Plant authentication and collection

*Rorippa islandica* (Oeder ex Murr.) and *Carrichtera annua* L. aerial parts (Family: Brassicaceae) were collected in April–May 2022 from the NorthWestern Coastal region (Marsa Matrouh, Egypt) at the flowering stage. The study site was selected based on several scientific and logistical considerations, including: natural abundance of target species for representative sampling, accessibility and safety for logistical feasibility, and minimal pollution/human interference to ensure data reliability.

The plant specimens were authenticated at the Herbarium by Dr. Omran Ghaly, Researcher at the Plant Ecology Department, Desert Research Center, Cairo, Egypt. The assigned codes were: *R. islandica* (Oeder ex Murr.) (CAIH-1258-RPH) and *C. annua* L. (CAIH-1084-R). The experimental and plant collection protocol was approved by the “Desert Research Center, Cairo, Egypt” (protocol serial number: MP 1841), and all procedures complied with relevant institutional, national, and international guidelines and legislation.

### Preparation and extraction

Each plant sample was air-dried at room temperature and ground into a fine powder using an electric grinder^15^. Thirty grams of powdered material from each plant were soaked separately in 70% ethanol at room temperature in flasks and shaken for 72 h. The suspensions were then filtered and concentrated under reduced pressure using a rotary evaporator (BűCHI VAC V-500) at 40 °C, followed by drying in an oven at 40 °C^16^.

### Fractionation by different organic solvent

Fifty-two grams of fine powder of each plant were put in the thimble and extracted successively three times with hexane, diethyl ether, chloroform, ethyl acetate, ethyl alcohol (96%) and ethyl alcohol (70%) for 72 h. All solvent extracts were condensed using a rotary evaporator. The fractions were preserved in air tight brown bottle until further use^17^.

### Gas Chromatography-Mass spectrometry (GC-MS)

The total ethanol extracts of *R. islandica* (Oeder ex Murr.) and *C. annua* L. aerial parts were subjected to GC-MS analysis to identify potent volatile and semi-volatile components. Each ethanol extract, dissolved in chloroform, was analyzed using a Trace GC1310-ISQ mass spectrometer (Thermo Scientific, Austin, TX, USA) equipped with a direct capillary column TG–5MS (30 m × 0.25 mm × 0.25 μm film thickness).The oven temperature was initially set at 35 °C, then increased at a rate of 3 °C/min to 200 °C (held for 3 min), and finally raised to 280 °C at 3 °C/min with a 10 min hold. The MS transfer line and injector were maintained at 250 °C and 260 °C, respectively. Helium was used as the carrier gas at a constant flow rate of 1 mL/min.

After a three-minute solvent delay, diluted samples (1 µL) were automatically injected using an Autosampler AS1300 in split mode. In full-scan mode, EI mass spectra were collected at 70 eV ionization voltage over the m/z range of 40–1000. The ion source temperature was set at 200 °C. Compound percentages were calculated based on relative peak area normalization of the GC-MS chromatogram and identified by comparing their mass spectra and retention times with those in the NIST11 and WILEY 09 mass spectral libraries^18^.

### Qualitative and quantitative determination of phenolic compounds using high performance liquid chromatography (HPLC) technique

HPLC analysis of the 70% ethanol total extract was carried out using an Agilent 1260 series system. Separation was achieved on a Zorbax Eclipse Plus C8 column (4.6 mm × 250 mm, 5 μm). The mobile phase consisted of water (A) and 0.05% trifluoroacetic acid in acetonitrile (B), delivered at a flow rate of 0.9 mL/min. The gradient program was as follows: 82% A at 0 min; 82% A from 0 to 1 min; 75% A from 1 to 11 min; 60% A from 11 to 18 min; 82% A from 18 to 22 min; and 82% A from 22 to 24 min. Detection was performed at 280 nm using a multi-wavelength detector. The injection volume was 5 µL, and the column temperature was maintained at 40 °C^19^.

### Fungal strains

*Alternaria cerealis* (MT808477), *Fusarium solani* (OK464437), *Curvularia lunata* (OM432028), *Penicillium glabrum* (OP694171), and *Colletotrichum gloeosporioides* (OP177948) were the standard fungal cultures used in this study. These strains were obtained from the Moubasher Mycological Center (AUMMC), Assiut University.

### Media

Potato dextrose agar (PDA) medium (g/L) was prepared according to the manufacturer’s instructions by dissolving 39 g of PDA powder in 1 L of distilled water. The mixture was stirred until completely dissolved, then autoclaved at 121 °C for 15 min. After sterilization, approximately 25 mL of medium was poured into each Petri dish^20^.

Potato dextrose broth (PDB) medium (Liofilchem, Italy) was prepared by suspending 27 g of the medium in 1 L of distilled water and warming gently until fully dissolved. The medium was sterilized by autoclaving at 121 °C for 15 min^20^.

Sabouraud’s broth medium (g/L) was prepared with glucose (20.0), peptone (10.0), KH2PO4 (1.0), and MgSO4·7H2O (1.0), adjusted to pH 6.5^21^.

### Standard drugs

The antifungal drug used as a control in this study was Benozed 25%, obtained from Kafr El Zayat for Pesticides & Chemicals, Egypt. It was dissolved in distilled water.

### Antifungal activity

All plant pathogenic fungi were cultivated on Potato Dextrose Agar (PDA) medium using the agar well diffusion method, which is commonly employed to evaluate the antimicrobial properties of plants. After preparation, the agar plates were inoculated with fungal suspensions within 15 min. The fungal strains were cultured on PDA medium at 25 °C. Using a sterile loop, sporulated fungi were removed from agar slants and suspended in 10 mL of sterile water to prepare the inocula. To eliminate hyphae, the suspensions were filtered through sterile gauze. The resulting conidial suspensions were vortexed thoroughly, and sterile distilled water was added to adjust the concentration to 10⁵ CFU/mL.

The dried agar surface was evenly streaked in three directions and allowed to dry for no more than 15 min. Using a sterile cork borer or tip, wells of 6–8 mm diameter were aseptically punched in the agar. A volume of 100 µL of each extract solution at the required concentration (10 mg/mL) was added to the wells. After adding the extract, plates were refrigerated within 15 min. Following 48–72 h of incubation, the diameters of inhibition zones (in mm) around the wells were measured at the point where growth was clearly reduced^22^. All experiments were performed in triplicate.

### Determination of minimum inhibitory concentration (MIC) for pathogenic fungi

MIC values were determined using the broth microdilution technique for extracts or solvent fractions^23^. This method is referred to as “microdilution” because it employs small volumes of broth dispensed in sterile plastic microdilution trays with conical or round-bottom wells, each containing 0.1 mL of broth.

Serial two-fold dilutions of the antifungal agents were prepared volumetrically in broth. A single pipette was used to measure all diluents and to add the stock antifungal solution to the first tube; new pipettes were used for each subsequent dilution step. The antifungal/broth solutions were dispensed into 96-well microdilution trays, delivering 0.1 (± 0.02) mL per well.

The fungal strains were cultivated on Sabouraud Dextrose broth medium at 25 °C. Sporulated fungi were collected from agar slants using a sterile loop and suspended in 10 mL of sterile water to prepare the inocula. The suspensions were filtered once through sterile gauze to remove hyphae, then vortexed vigorously. Using a hemocytometer, the conidial suspensions were adjusted with sterile distilled water to 10⁵ CFU/mL, confirmed by serial dilution plate counts. These suspensions were further diluted (1:5) in Sabouraud Dextrose broth to obtain 2× final suspensions. When mixed with antifungal solutions, the final concentration of conidia was 10⁴ CFU/mL.

The prepared conidial suspensions were inoculated into 96-well plates and incubated at 35 °C for 48 h. MICs were recorded at 24 and 48 h as the lowest extract concentration that resulted in complete growth inhibition compared to the extract-free control wells^24^.

### Determination of minimum fungicidal concentration (MFC)

MFC values were determined by subculturing 10 µL from wells of the microdilution plates that corresponded to or exceeded the lowest MIC values, onto Sabouraud Dextrose broth medium, followed by incubation for 24 h. After incubation, the Petri dishes were examined for fungal growth. The lowest concentration of extract that showed no visible growth was recorded as the MFC^25^. All experiments were performed in triplicate.

### Determination of ergosterol content in the plasma membrane using HPLC technique

Total intracellular sterols were extracted as described by^26^ with slight modifications. Briefly, spores from 48 to 72 h PDA plate cultures were used to inoculate 50 mL of potato dextrose broth containing sub-MIC concentrations of each extract. Cultures were incubated at 25 °C with shaking for 72 h. Cells were harvested by centrifugation at 3000 rpm for 5 min, washed with sterile distilled water, and the wet weight of the pellet was recorded.

Each pellet was mixed with 3 mL of 25% alcoholic potassium hydroxide solution (25 g KOH and 35 mL distilled water, adjusted to 100 mL with 100% ethanol) and vortexed for 1 min. The suspensions were transferred to glass screw-cap tubes and incubated in a water bath at 85 °C for 1 h, then cooled to room temperature. A mixture of 1 mL distilled water and 3 mL n-heptane was added, followed by vigorous vortexing for 3 min to extract sterols. The heptane layer was transferred to glass screw-cap tubes and stored at − 20 °C for up to 24 h.

Before analysis, 20 µL of sterol extract was diluted fivefold in 100% ethanol. HPLC analysis was performed using an Agilent system with a detection wavelength of 280 nm, connected to Data Station Software. Chromatographic separation was achieved on a C18 column (250 × 4.6 mm, 5 μm). The mobile phase consisted of 98% methanol, delivered at a flow rate of 1.0 mL/min. The injection volume was 20 µL, and the column temperature was maintained at 25 °C^26,27^.

### Microscopic study of fungal morphology

Over the past decade, the use of three-dimensional imaging techniques to study the architecture of cell surfaces under physiological conditions has gained increasing attention. These techniques allow visualization of the outermost cell surface at molecular or nanoscale resolution, enabling direct observation of cell wall components^28^.

Atomic Force Microscop (AFM)^29^, a type of scanning probe microscopy (SPM), provides resolution at the nanometer scale, more than 1000 times higher than the optical diffraction limit. AFM data are obtained using a mechanical probe that “feels” the surface. Precise scanning is achieved by piezoelectric components that allow extremely small, accurate movements under electronic control. Despite its name, AFM does not rely on nuclear forces.

The morphological changes induced by the total and successive ethanol extracts of *R. islandica* (Oeder ex Murr.) and *C. annua* L. on the tested fungi were examined using AFM^30,31^. All images were captured in contact mode using a wet model AFM (SPM 9600, Shimadzu, Japan, Non-contact mode). The experiments were conducted at the Microanalytical Center, Cairo University.

## Results

### Gas Chromatography-Mass spectrometry (GC-MS)

**Fig. 1:**
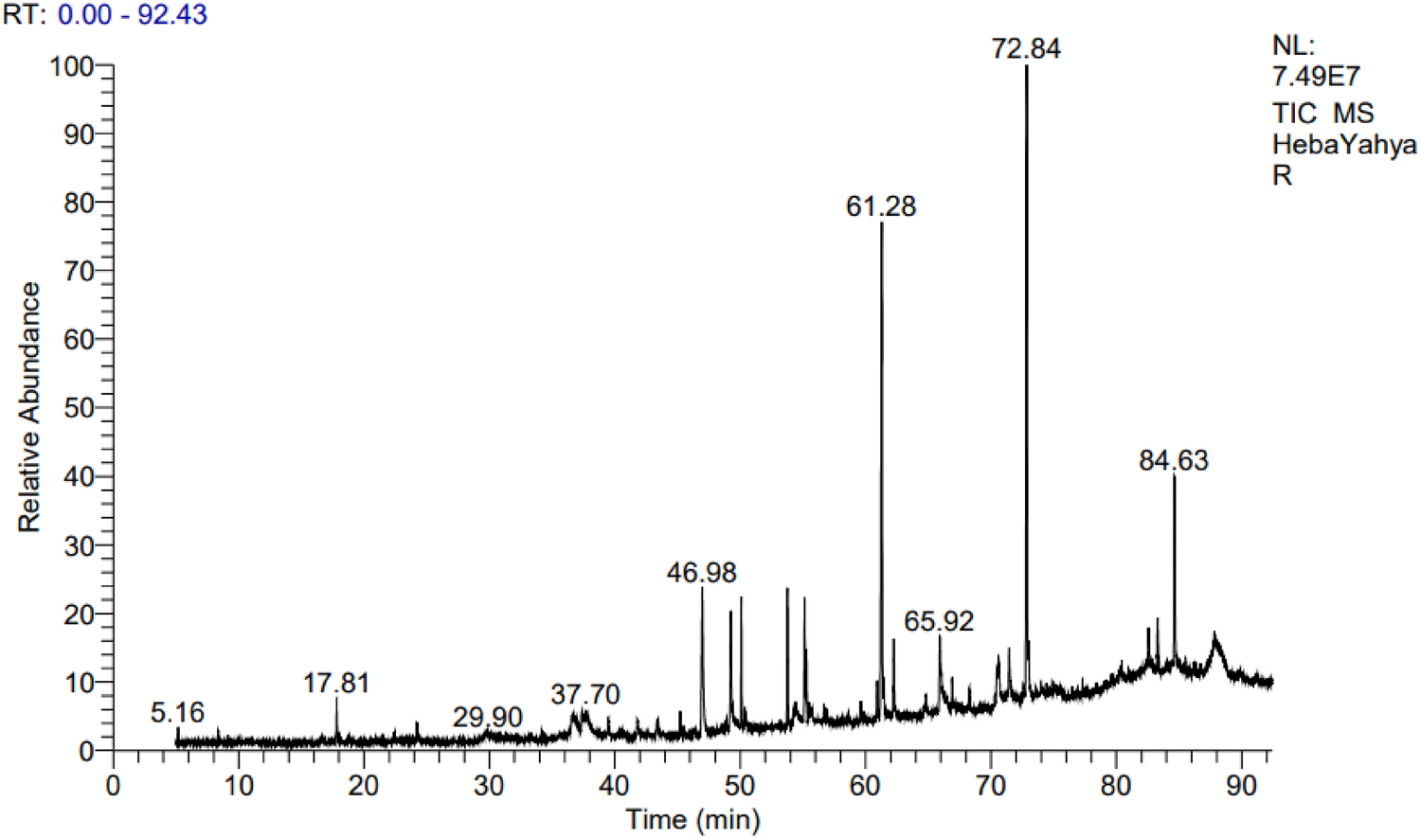
GC-MS analysis of *R. islandica* (Oeder ex Murr.)

**Fig. 2:**
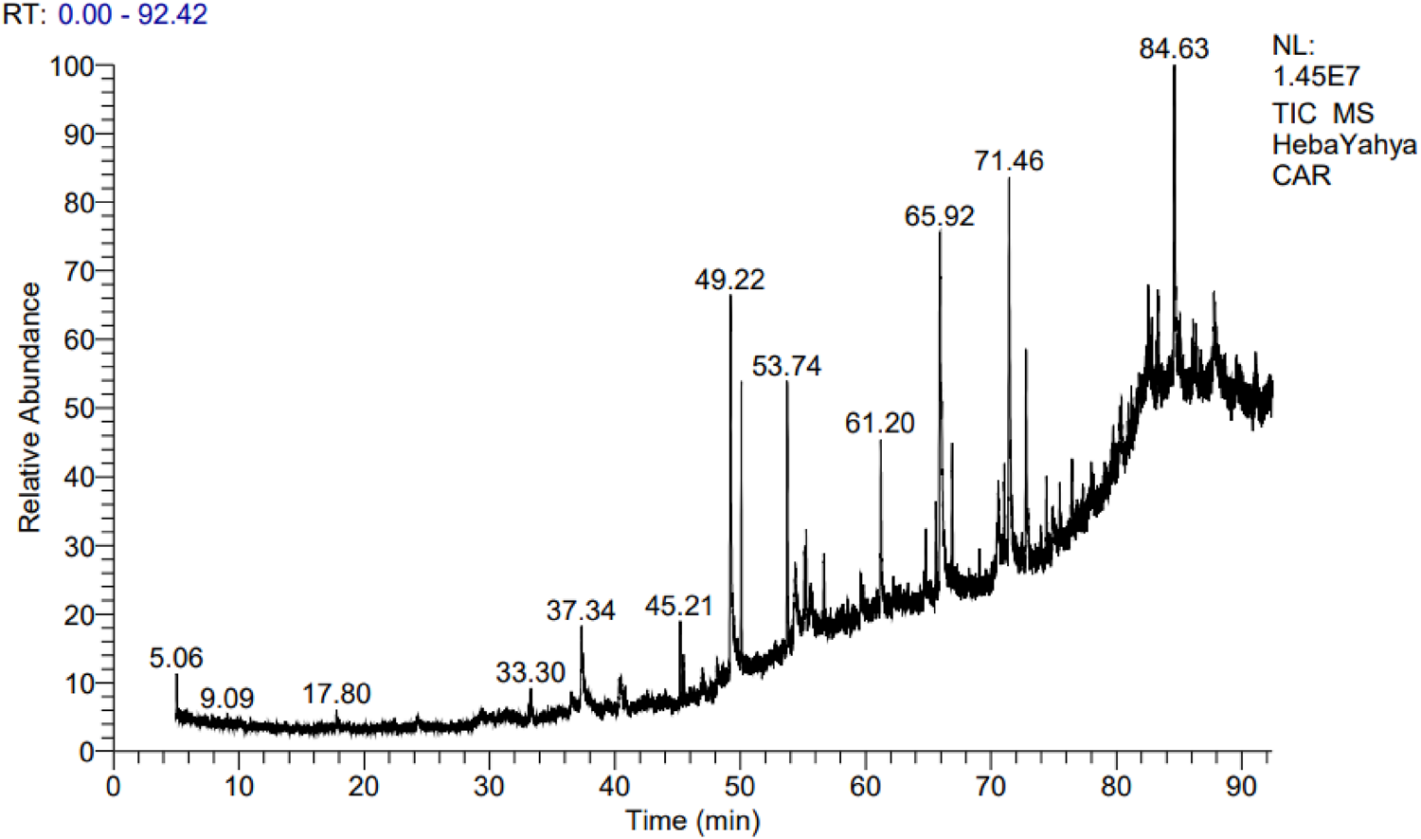
GC-MS analysis of ethanol extract of *C. annua* L.

GC-MS analysis was used to identify the chemical constituents of the total ethanol extracts of *R. islandica* (Oeder ex Murr.) and *C. annua* L. Based on their retention times, the main compounds (those with the highest relative percentage abundance in the sample) are presented in Tables 1 and 2; Figs. 1 and 2.

For *R. islandica* (Oeder ex Murr.), GC-MS revealed 50 compounds in the total ethanol extract. The major constituents were 13-Docosenamide (Z) (20.54%), 9-Octadecenamide (16.60%), β-Sitosterol (6.10%), Bicyclo (8.2.0) dodecane, 11, 11-dimethyl (5.85%), and Hexadecanoic acid (3.81%) (Table 1).

For *C. annua* L., the total ethanol extract contained 54 compounds. The principal constituents were 2-Hydroxy-1-(hydroxymethyl) ethyl stearate (9.74%), Glycerol 1-palmitate (8.59%), Hexadecanoic acid (8.55%), Stigmast-5-en-3-ol (6.98%), Phytol (3, 7, 11, 15-tetramethylhexadec-2-en-1-ol) (4.64%) and Pregn-5-ene-3,11-dione, 17,20:20,21-bis[methylenebis(oxy)]-, cyclic 3-(1,2-ethanediyl acetal) (4.03%) (Table 2).

### Identification of phenolic compounds using HPLC

**Fig. 3:**
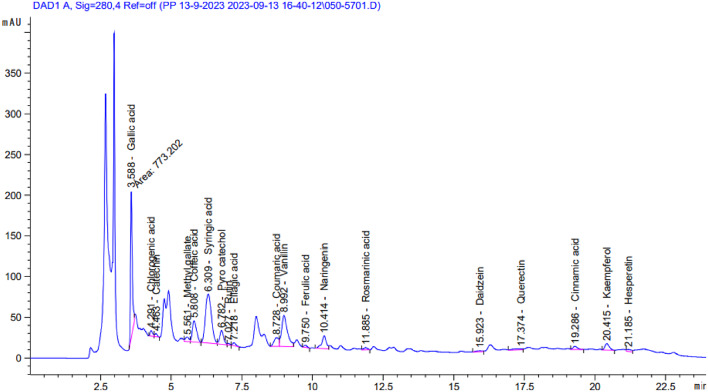
HPLC analysis of ethanol extract of *R. islandica* (Oeder ex Murr.)

**Fig. 4:**
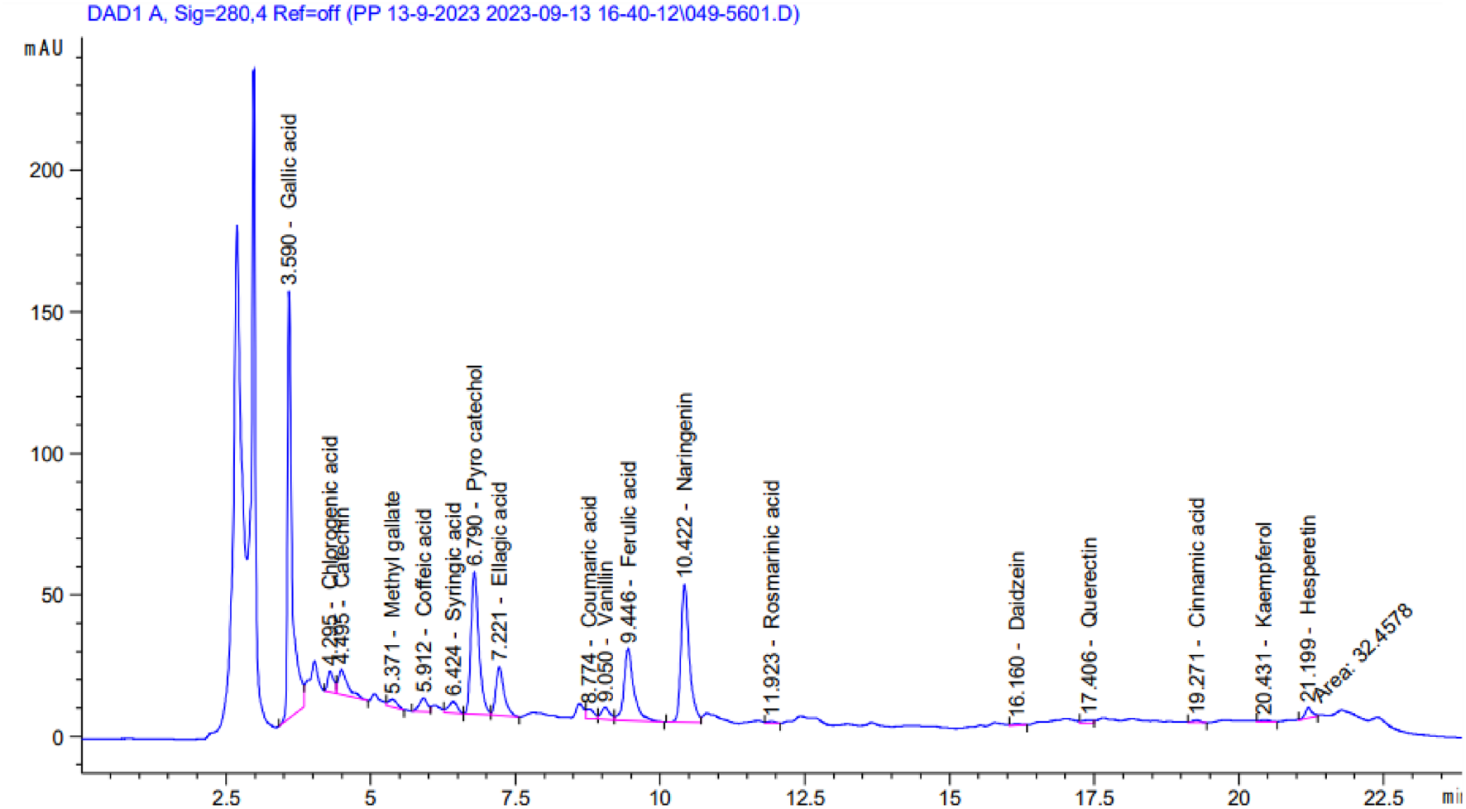
HPLC of ethanol extract of *C. annua* L.

The phenolic constituents of the ethanol extracts of *R. islandica* (Oeder ex Murr.) and *C. annua* L. were identified and quantified using HPLC (Table 3; Figs. 3 and 4).Data showed that gallic acid was the major phenolic compound in both plants, with concentrations of 3417.72 µg/g in *R. islandica* and 3733.98 µg/g in *C. annua*. In contrast, cinnamic acid was the minor compound in both plants, with concentrations of 36.74 µg/g and 7.82 µg/g, respectively.

In *R. islandica*, the main compounds were gallic acid, syringic acid, pyrocatechol and coffeic acid with concentrations of 3417.72 µg/g, 3326.94 µg/g, 1182.12 µg/g and 1101.98 µg/g respectively. In *C. annua*, the predominant compounds were gallic acid, pyrocatechol, naringenin and Catechin with concentrations of 3733.98 µg/g, 3511.85 µg/g, 2356.00 µg/g and 1153,44 respectively.Rutin was detected in *R. islandica* (75.02 µg/g) but was not detected in *C. annua*.

**Table 1:** GC-MS analysis of ethanol extract of *R. islandica (*Oeder ex Murr.)

RT (min)	Compound name	Chemical formula	MW	Area %
5.17	Dimethoxypropane	C_5_H_12_O_2_	104	0.21
8.36	1,5-Hexadien-3-ol	C_6_H_10_O	98	0.27
9.13	S-Methyl methanethiosulfinate	C_2_H_6_OS_2_	110	0.16
17.81	8-Nonene-1-nitrile	C_9_H_15_N	137	1.10
22.43	9-Decene-1-nitrile	C_10_H_17_N	151	0.25
24.20	2-Methoxy-4- vinylphenol	C_9_H_10_O_2_	150	0.62
34.19	2,5-Dimethoxy-4- ethylamphetamine	C_13_H_21_NO_2_	223	0.36
36.58	Lactose	C_12_H_22_O_11_	342	0.95
36.74	Ethyl à-d- glucopyranoside	C_8_H_16_O_6_	208	0.96
37.36	D-Glucitol, 1-S- hexyl-1-thio-	C_12_H_26_O_5_S	282	0.58
37.75	Desulphosinigrin	C_10_H_17_NO_6_S	279	0.80
39.48	4-Chloro-2,5- Dimethoxyamphetamine	C_11_H_16_ClNO_2_	229	0.60
41.80	1 H-Indole-3- acetonitrile	C_10_H_8_N_2_	156	0.78
43.35	8-Methyl-8-azabicyclo [3.2.1]oct-3-yl benzoate	C_15_H_19_NO_2_	245	0.85
45.23	2-Pentadecanone, 6,10,14-trimethyl-	C_18_H_36_O	268	0.53
46.98	Bicyclo [8.2.0] dodecane, 11,11-dimethyl-	C_14_H_26_	194	5.85
48.88	2-Aminoethanethiol hydrogen sulfate (ester)	C_2_H_7_NO_3_S_2_	157	0.41
49.25	Hexadecanoic acid	C_16_H_32_O_2_	256	3.81
50.10	Palmitic acid, ethyl ester	C_18_H_36_O_2_	284	2.79
50.37	Ethanol, 2-(9- octadecenyloxy)-, (z)-	C_20_H_40_O_2_	312	0.79
53.76	Phytol	C_20_H_40_O	296	3.69
54.40	Linolenin, 1-mono-	C_21_H_36_O_4_	352	1.57
55.14	9-Octadecenamide, 12-hydroxy-, [R-(Z)]-	C_18_H_35_NO_2_	297	3.68
55.28	Linolenic acid, ethyl ester	C_20_H_34_O_2_	306	2.26
55.50	9-Octadecenoic acid (Z)-, ethyl ester (Ethyl oleate)	C_20_H_38_O_2_	310	0.50
55.73	Oleic acid	C_18_H_34_O_2_	282	0.47
56.69	Stearic acid, ethyl ester	C_20_H_40_O_2_	312	0.47
56.86	2,3,4,5- Tetrahydroxypentanal	C_5_H_10_O_5_	150	0.45
58.61	2,3-Dihydroxypropyl palmitate	C_19_H_38_O_4_	330	0.32
59.61	1,1-Diphenyl-1-(2- dimethylaminoethyl)-2-butanone	C_20_H_25_NO	295	0.55
60.93	9,12-Octadecadienoic acid (z, z)-, 2,3-dihydroxypropyl ester	C_21_H_38_O_4_	354	1.18
61.28	9-Octadecenamide	C_18_H_35_NO	281	16.60
62.24	Stearamide (octadecanamide)	C_18_H_37_NO	283	2.22
64.82	Di-2-Benzothiazole disulfane	C_14_H_8_N_2_S_4_	332	0.61
65.93	Palmitin, 2-mono-	C_19_H_38_O_4_	330	2.89
66.91	1,2-Benzenedicarboxylic acid	C_24_H_38_O_4_	390	0.75
68.27	2,3-Dihydroxypropyl stearate	C_21_H_42_O_4_	358	0.78
70.30	Propanoic acid, 2- (3-acetoxy-4,4,14- trimethylandrost-8- en-17-yl)	C_27_H_42_O_4_	430	0.15
70.45	Ethyl iso- allocholate	C_26_H_44_O_5_	436	0.93
70.59	1,25-Dihydroxyvitamin D3, TMS derivative	C_30_H_52_O_3_Si	488	2.14
71.45	Distearin	C_39_H_76_O_5_	624	1.70
72.84	13-Docosenamide, (z)-	C_22_H_43_NO	337	20.54
73.98	6,8-DI-C-á- Glucosylluteolin	C_27_H_30_O_16_	610	0.75
74.96	Pregn-5-ene-3,11- dione, 17,20:20,21- bis[methylenebis(oxy)]-, cyclic 3-(1,2-ethanediyl acetal)	C_25_H_34_O_7_	446	0.64
82.57	Campesterol	C_28_H_48_O	400	1.41
83.29	Stigmasterin	C_29_H_48_O	412	1.72
84.64	Ç-Sitosterol	C_29_H_50_O	414	6.10
85.49	7,8-Epoxylanostan- 11-ol, 3-acetoxy-	C_32_H_54_O_4_	502	0.51
86.14	3’,4’,7-Trimethylquercetin	C_18_H_16_O_7_	344	0.49
87.81	Stearin, 1,3- dipalmito-2-	C_53_H_102_O_6_	834	1.22

RT: Retention Time. Mw: Molecular weight.

**Table 2:** GC-MS analysis of ethanol extract of *C. annua* L.

RT (min)	Compound name	Chemical formula	MW	Area %
5.06	Propionaldehyde, dimethyl acetal	C_5_H_12_O_2_	104	0.33
17.80	8-Nonene-1-nitrile	C_9_H_15_N	137	0.31
33.10	Acetone (1r)-(+)- camphor azine	C_13_H_22_N_2_	206	0.27
33.30	3-(N, N-Dimethyllaurylammonio) propanesulfonate	C_17_H_37_NO_3_S	335	0.62
36.53	Estra-1,3,5(10)- trien-17á-ol	C_18_H_24_O	256	0.65
37.33	A-mannopyranoside-1- methyl-2,3–4,6-di-butylboronate	C_15_H_28_B_2_O_6_	326	1.96
40.34	4-((1E)-3-Hydroxy-1-propenyl)-2- methoxyphenol	C_10_H_12_O_3_	180	0.64
40.50	4-(1-Hydroxy-2-isopropyl-5- methylcyclohexyl)-3-butyn-2-one	C_14_H_22_O_2_	222	0.72
40.83	2-Acetyl-3-(2-cinnamido) ethyl-7-methoxyindole	C_22_H_22_N_2_O_3_	362	0.42
43.98	Oleic acid	C_18_H_34_O_2_	282	0.62
45.21	2-Pentadecanone, 6,10,14-trimethyl	C_18_H_36_O	268	1.23
45.46	2-Cis-9- Octadecenyloxyethanol	C_20_H_40_O_2_	312	0.73
46.83	Stearic acid	C_18_H_36_O_2_	284	0.24
46.99	2-Aminoethanethiolsulfuric acid	C_2_H_7_NO_3_S_2_	157	0.72
47.89	Methyl 16-hydroxy- hexadecanoate	C_17_H_34_O_3_	286	0.24
48.14	Linolenin, 1-mono-	C_21_H_36_O_4_	352	0.74
49.23	Hexadecanoic acid	C_16_H_32_O_2_	256	8.55
50.08	Palmitic acid, ethyl ester	C_18_H_36_O_2_	284	3.80
53.75	3,7,11,15-Tetramethylhexadec- 2-en-1-ol (phytol)	C_20_H_40_O	296	4.64
54.38	Methyl 8-[2-((2-[(2-ethyl cyclopropyl)methyl] cyclopropyl)methyl) cyclopropyl]octanoate	C_22_H_38_O_2_	334	2.86
55.12	[1,1’-Bicyclopropyl]-2- octanoic acid, 2’-hexyl-, methyl ester	C_21_H_38_O_2_	322	1.47
55.26	Linolenic acid, ethyl ester	C_20_H_34_O_2_	306	1.67
55.62	Aqua cera	C_22_H_44_O_4_	372	0.81
56.67	Ethyl octadecanoate	C_20_H_40_O_2_	312	1.38
58.57	Di-2-benzothiazole disulfane	C_14_H_8_N_2_S_4_	332	1.06
59.60	Pentadecanoic acid	C_15_H_30_O_2_	242	1.17
59.82	Dasycarpidan-1- methanol, acetate (ester)	C_20_H_26_N_2_O_2_	326	0.64
60.91	Stearin, 1,3-di-	C_39_H_76_O_5_	624	0.32
61.21	9-Octadecenamide	C_18_H_35_NO	281	3.56
65.60	Methyl 8-(7-hexyl- 3,7-dihydro-4a (4 H)-naphthalenyl) octanoate	C_25_H_40_O_2_	372	1.65
65.92	Glycerol 1- palmitate	C_19_H_38_O_4_	330	8.59
66.90	3’,8,8’-Trimethoxy- 3-piperidin-1-yl- 2,2’-binaphthyl- 1,1’,4,4’-tetrone	C_28_H_25_NO_7_	487	2.22
71.45	2-Hydroxy-1- (hydroxymethyl) ethyl stearate	C_21_H_42_O_4_	358	9.74
72.79	18,19-Secoyohimban- 19-oic acid,16,17,20,21- tetradehydro-16-(hydroxymethyl)-, methyl ester, (15á,16E)-	C_21_H_24_N_2_O_3_	352	4.03
72.85	6,8-DI-C-á- Glucosylluteolin	C_27_H_30_O_16_	610	3.26
74.95	Pregn-5-ene-3,11- dione, 17,20:20,21- bis[methylenebis(oxy)]-, cyclic 3-(1,2- ethanediyl acetal)	C_25_H_34_O_7_	446	1.28
75.48	N-(2-{4,5-dimethoxy- 2-[2-phenylethenyl] phenyl }-3-phenylpropyl)-n, ndimethylaminehydrochloride	C_27_H_32_ClNO_2_	437	0.88
76.45	3’,4’,7- Trimethylquercetin	C_18_H_16_O_7_	344	3.13
80.26	Cholest-5-en-3-ol (3á)-	C_27_H_46_O	386	1.31
80.96	1,5-Dimethoxy-2,4- bis(3-methylphthalidyl) benzol	C_26_H_22_O_6_	430	1.09
81.20	3-(Tetradecanoyloxy)- 2-[(trimethyl) oxy] propyl myristate	C_34_H_68_O_5_	584	0.81
81.49	Docosanoic acid, 1,2,3-propanetriyl ester	C_69_H_134_O_6_	1058	0.13
81.81	Flavone 5,7- oh,3’,4’-ome	C_17_H_14_O_6_	314	0.51
82.34	3-Hydroxyspirost- 8-en-11-one	C_27_H_40_O_4_	428	0.38
82.55	Ethyl 3,7,12- trihydroxycholan-24-oate	C_26_H_44_O_5_	436	2.44
82.83	Palmitin, 1,2-di-	C_35_H_68_O_5_	568	0.92
84.40	3-[(Z)-2-Phenylethenyl] cholestan-2-one	C_35_H_52_O	488	0.31
84.63	Stigmast-5-en-3-ol	C29H_50_O	414	6.98
86.13	Methyl glycocholate, 3TMS derivative	C_36_H_69_NO_6_	695	1.76
86.73	7,8-Epoxylanostan- 11-ol, 3-acetoxy-	C_32_H_54_O_4_	502	0.87
87.80	Propanoic acid, 2-(3-acetoxy- 4,4,14-trimethylandrost-8-en-17-yl)-	C_27_H_42_O_4_	430	2.50
91.01	4a-phorbol-12, 13-didecanoat	C_40_H_64_O_8_	672	0.36
91.08	H-purin-6-amine, [(2-fluorophenyl) methyl]-	C_12_H_10_FN_5_	243	1.73
92.15	28 lidbpzoyraxili- qinsgfpzsa-n	C_32_H_39_NO_10_	597	0.27

RT: Retention Time. Mw: Molecular weight.

**Table 3:** Phenolic composition of ethanol extract of *R. islandica* (Oeder ex Murr.) and *C. annua* L. using HPLC technique.

RT. (min)	Compound name	*R*. *islandica* (Oeder ex Murr.)		*C. annua* L.	
		Area	Conc. µg/g	Area	Conc. µg/g
3.59	Gallic acid	773.20	3417.72	844.75	3733.98
4.29	Chlorogenic acid	43.95	297.10	52.63	355.73
4.46	Catechin	23.57	268.79	101.16	1153.44
5.56	Methyl gallate	52.80	136.69	27.31	70.69
5.81	Coffeic acid	269.62	1101.98	47.87	195.64
6.31	Syringic acid	887.37	3326.94	43.89	164.56
6.78	Pyro catechol	161.76	1182.12	480.55	3511.85
7.03	Rutin	9.35	75.02	N.D.	N.D.
7.22	Ellagic acid	27.73	121.98	171.65	755.08
8.73	Coumaric acid	122.32	225.86	31.96	59.01
8.99	Vanillin	552.28	1042.28	43.34	81.80
9.75	Ferulic acid	17.70	53.65	298.98	906.09
10.41	Naringenin	173.99	830.05	493.84	2356.00
11.89	Rosmarinic acid	25.69	140.45	7.31	39.95
15.92	Daidzein	16.43	47.58	5.22	15.13
17.37	Querectin	41.50	262.50	15.72	99.43
19.29	Cinnamic acid	39.79	36.74	8.47	7.82
20.42	Kaempferol	83.13	273.54	8.30	27.32
21.19	Hesperetin	28.45	73.18	32.46	83.51

N.D.: Not Detected.

### Antifungal study

#### Fungal strains

**Fig. 5:**
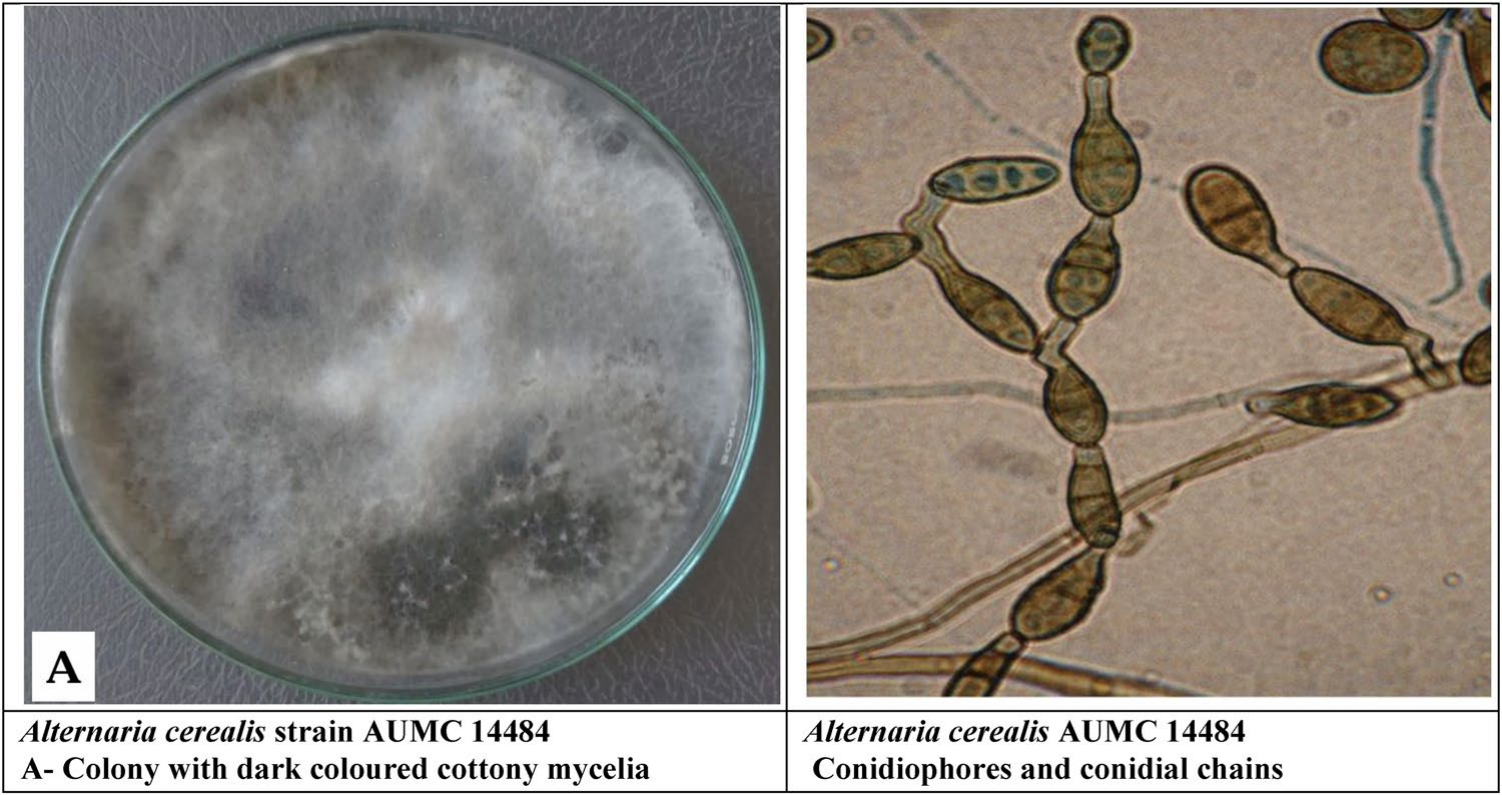
*Alternaria cerealis* (MT808477).

**Fig. 6:**
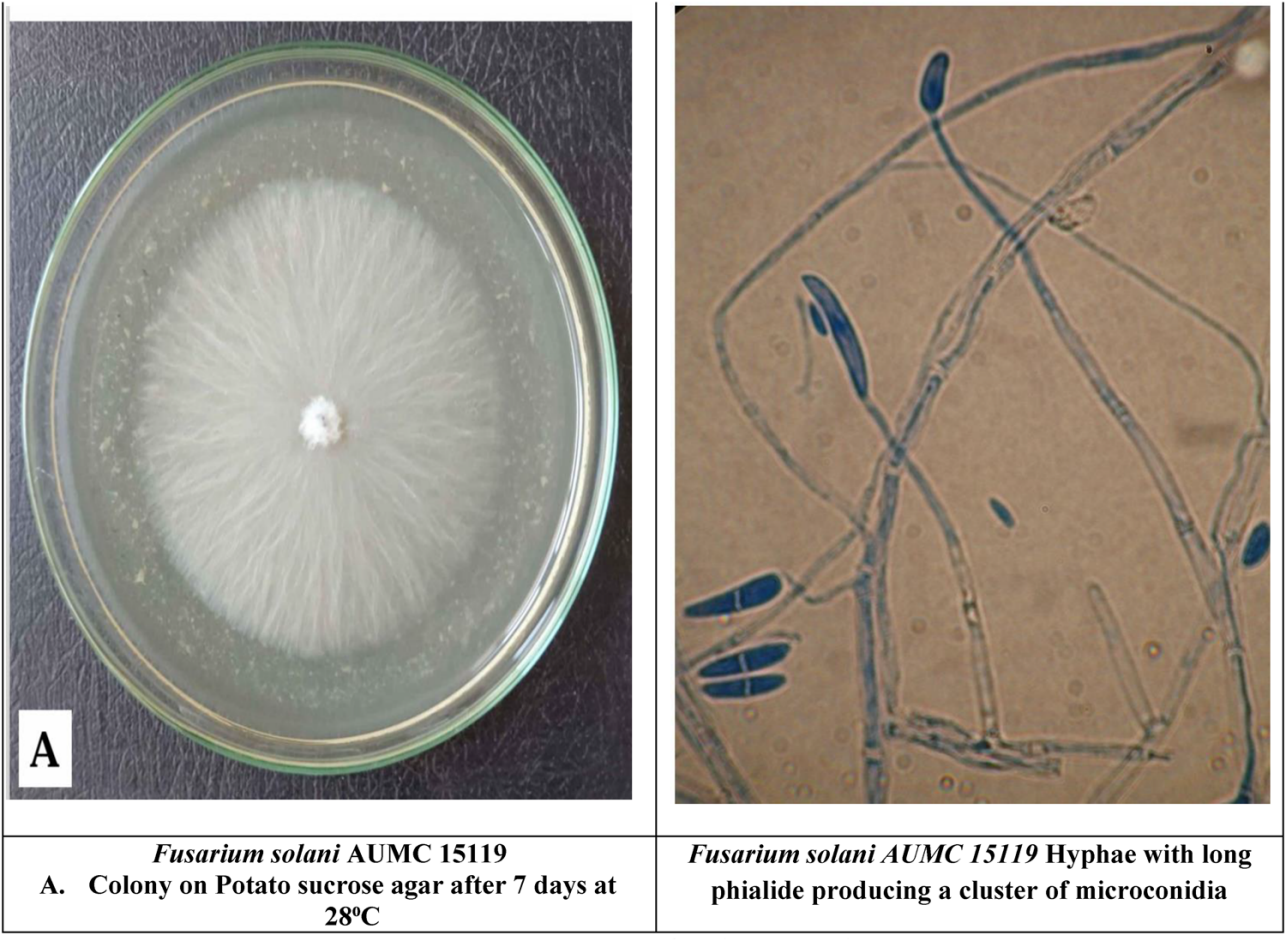
*Fusarium solani* (OK464437).

**Fig. 7:**
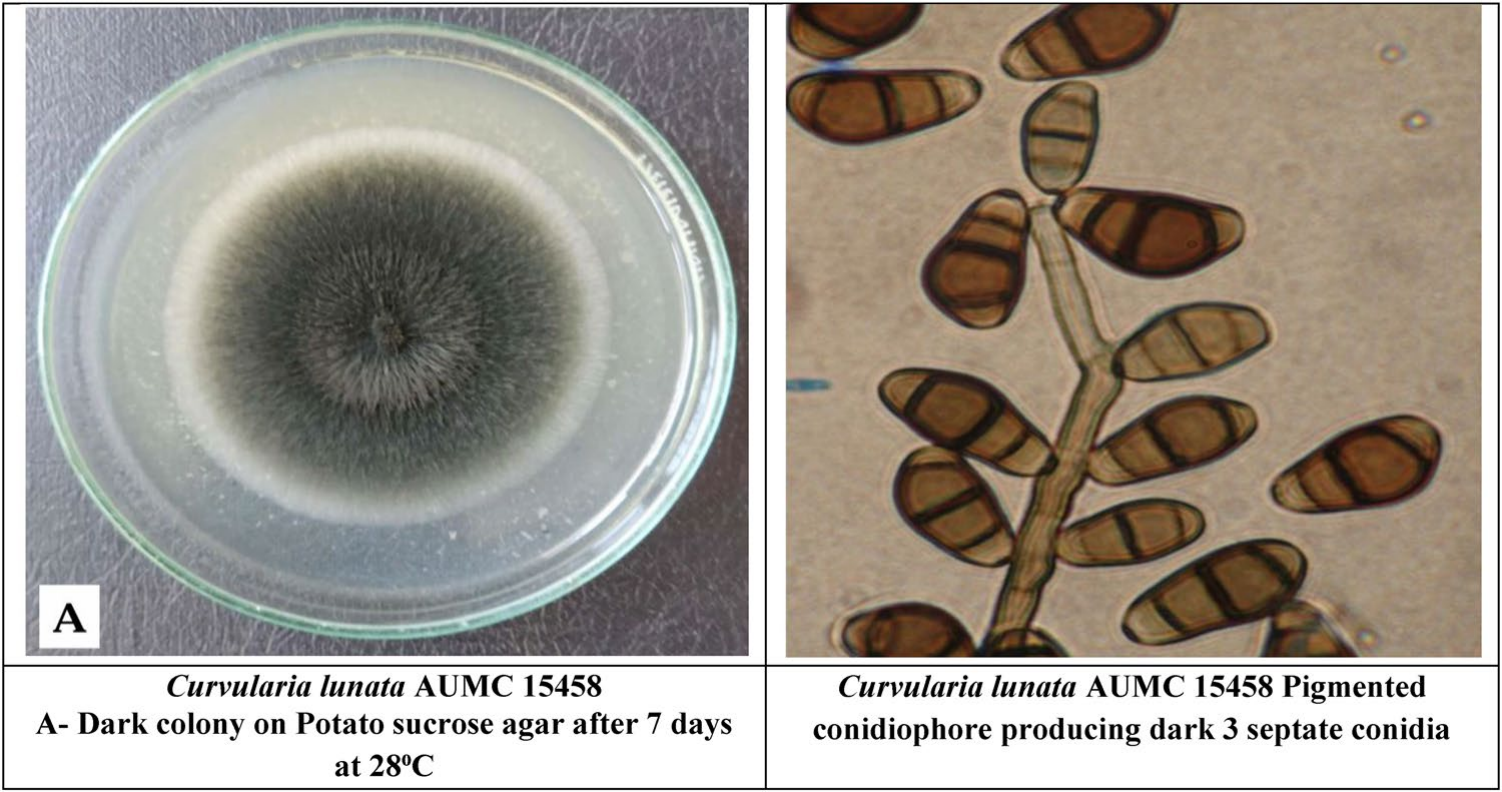
*Curvularia lunata* (OM432028).

**Fig. 8:**
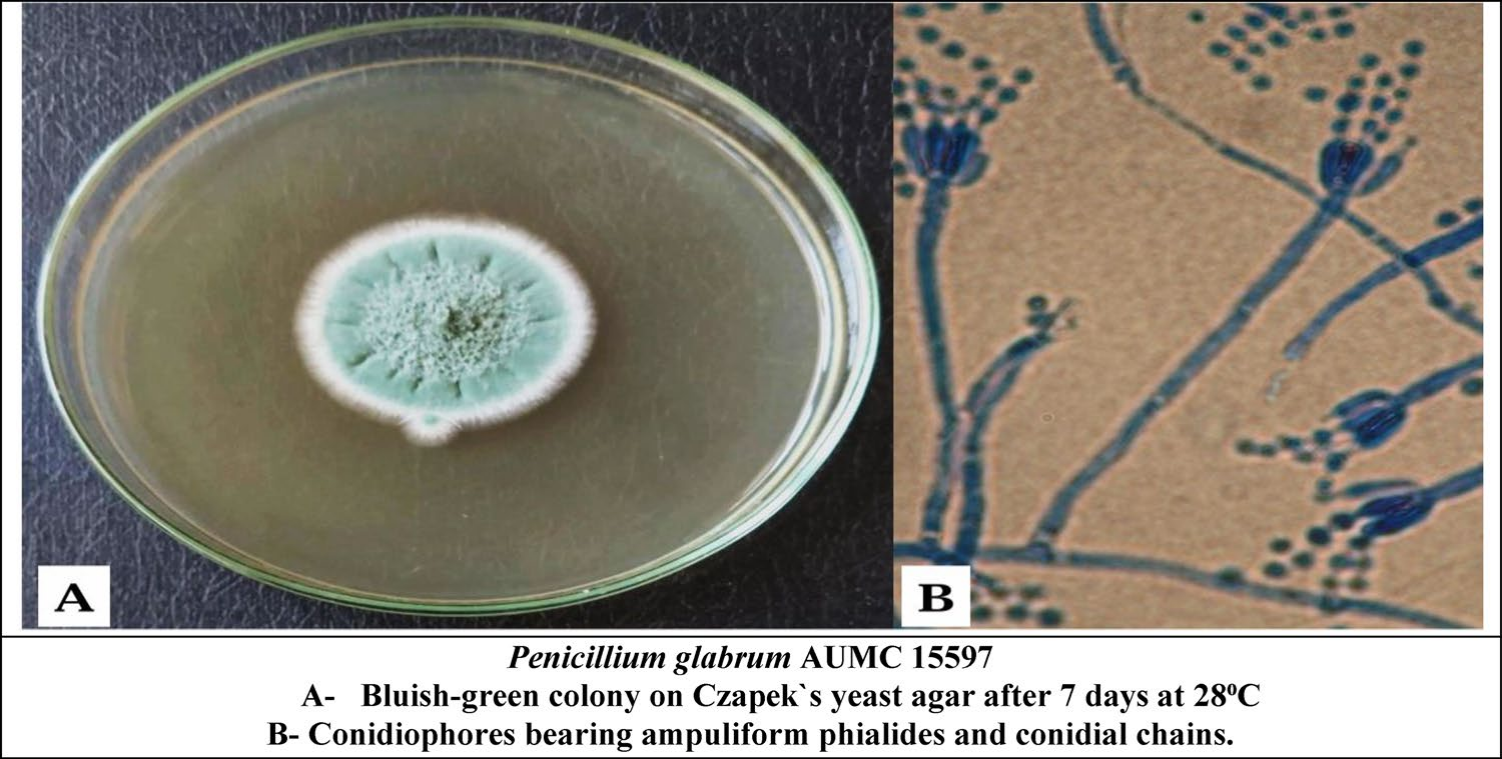
*Penicillium glabrum* (Op694171).

**Fig. 9:**
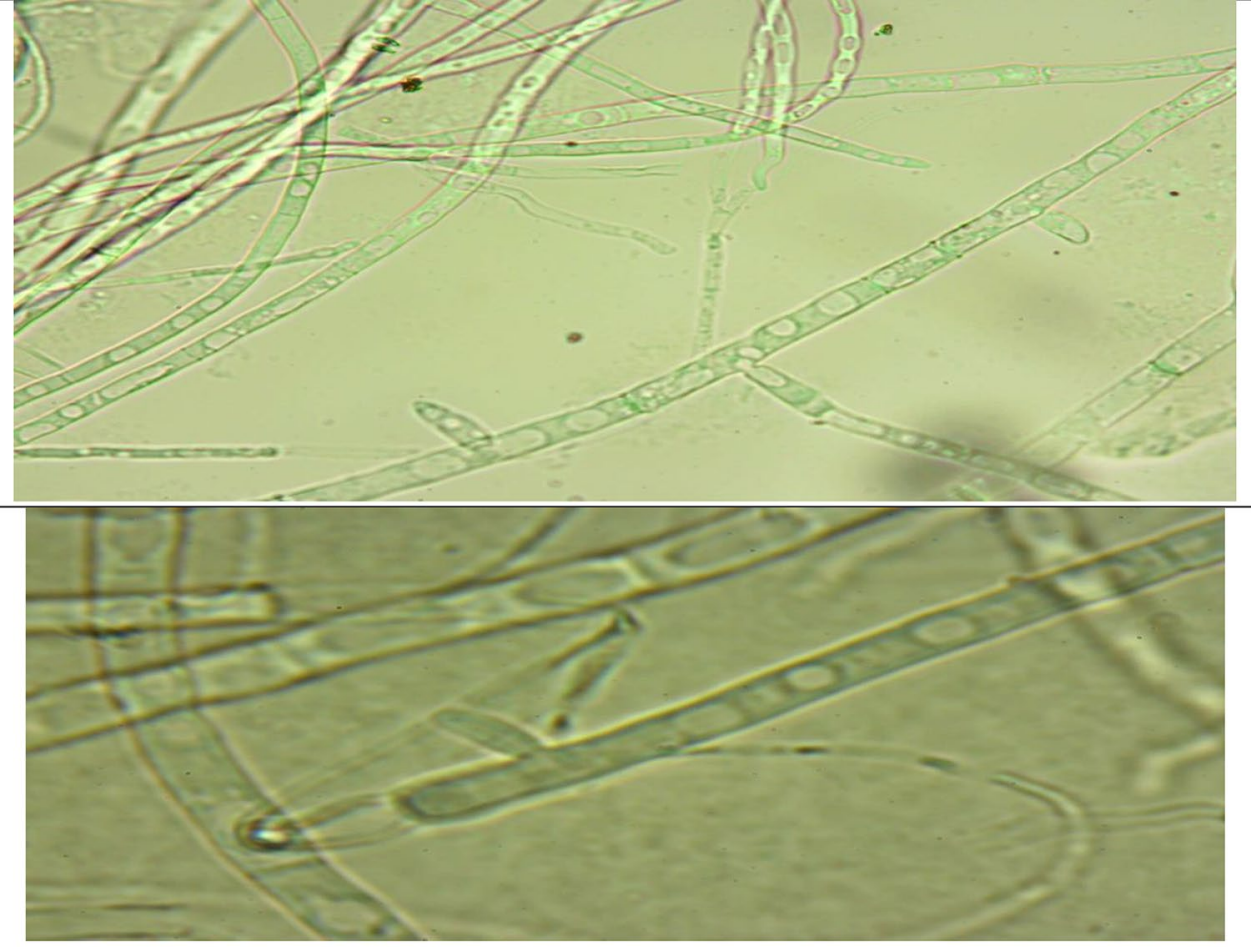
*Colletotrichum gloeosporioides* (OP177948).

The total crude extracts (70% ethanol) and successive extracts (hexane, diethyl ether, chloroform, ethyl acetate, 96% ethanol, and 70% ethanol) were tested against standard fungal cultures: *A. cerealis* (MT808477) (Fig. 5), *F. solani* (OK464437) (Fig. 6), *C. lunata* (OM432028) (Fig. 7), *P. glabrum* (OP694171) (Fig. 8), and *C. gloeosporioides* (OP177948) (Fig. 9).

#### Screening of antifungal activity using the agar diffusion method

The antifungal activity of the crude extracts of the two plants was assessed using inhibition zone diameters, with Benozed 25% as the positive control. Results indicated variable susceptibility among the tested fungi.

For *R. islandica* (Oeder ex Murr.), the total ethanol extract showed inhibitory effect against all tested fungi. The most sensitive species was *C. gloeosporioides* (inhibition zone 21 ± 0.1 mm), followed by *A. cerealis*, *C. lunata*, and *P. glabrum* (20 ± 0.1, 17 ± 0.1, and 16 ± 0.1 mm, respectively), while *F. solani* was the most resistant (12 ± 0.2 mm) (Table 4). The 96% ethanol extract was the most effective successive fraction, inhibiting all tested fungi. The 70% ethanol extract of *R. islandica* was active against *C. gloeosporioides*, *P. glabrum*, and *A. cerealis* (29 ± 0.3, 18 ± 0.2, and 18 ± 0.1 mm, respectively), but showed no activity against the other tested fungi.

For *C. annua* L., the total ethanol extract exhibited the strongest antifungal activity, with *C. lunata* being the most sensitive (35 ± 0.1 mm), followed by *C. gloeosporioides*, *A. cerealis*, and *P. glabrum* (28 ± 0.1, 22 ± 0.1, and 22 ± 0.2 mm, respectively). The weakest activity was observed against *F. solani* (17 ± 0.1 mm). The 70% ethanol extract of *C. annua* was active against *C. lunata*, *C. gloeosporioides*, *F. solani*, and *P. glabrum* (27 ± 0.1, 22 ± 0.3, 17 ± 0.2, and 15 ± 0.2 mm, respectively), but inactive against *A. cerealis*. Chloroform and ethyl acetate extracts of *C. annua* were active only against *A. cerealis* (19 ± 0.1 and 14 ± 0.1 mm, respectively).

**Table 4:**
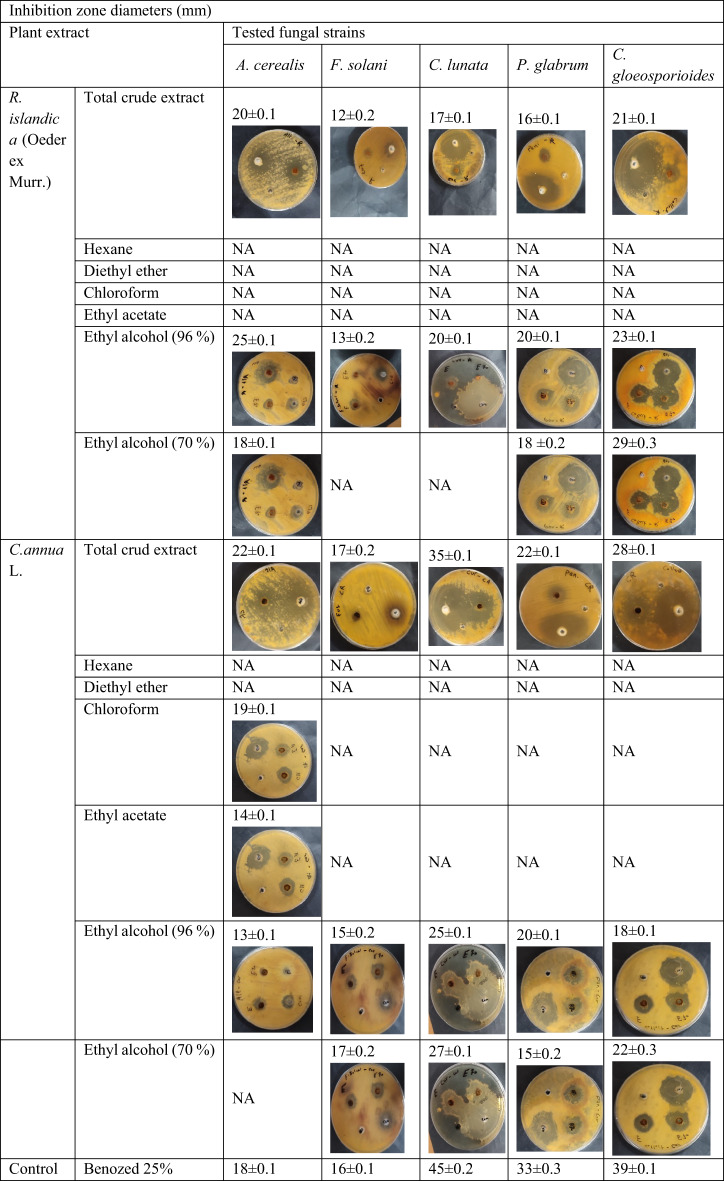
Antifungal activity of plant extracts against some tested fungal species using agar well diffusion method.

NA: No activity. ±: Standard deviation.

#### Determination of the minimum inhibitory concentration (MIC)

The MIC of most active plant extracts against tested fungal species was determined using broth microdilution assay. According to results showed at (Table 5) *C. annua* L. total ethanol extract has the lowest MIC value against *C. lunata* (1.97 mg/ml). Also, the lowest MIC value of successive extracts was for ethyl alcohol 70% of *R. islandica* (Oeder ex Murr.) against *C. gloeosporioides* (7.8 mg/ml) and also for ethyl alcohol 70% successive extract of *C. annua* L. against *C. lunata* (7.8 mg/ml).

#### Determination of the minimum fungicidal concentration (MFC)

Concerning the MFC, the two plant extracts and standard drug killed the tested fungal species at concentrations higher than MIC values. According to the results showed at (Table 5) *C. annua* L., total ethanol extract has the lowest MFC value against *C. lunata* (3.9 mg/ml), while ethyl alcohol (96%) of *R. islandica* (Oeder ex Murr.) successive extract had MFCs values ranging from (31.25–500 mg/ml). *A. cerealis* and *C. gloeosporioides* were the most sensitive tested fungi species followed by *C. lunata* and *P. glabrum*, then *F. solani*.

**Table 5:** MIC and MFC values of total and the most active plants extract on tested fungal species.

Samples		Activities	mg/ml				
			*A. cerealis*	*F. solani*	*C. lunata*	*P. glabrum*	*C. gloeosporioides*
*R. islandica* (Oeder ex Murr.)	Total crude extract	MIC	31.25	250.00	125.00	125.00	62.50
		MFC	62.50	1000.00	500.00	500.00	125.00
	Ethyl alcohol (96%)	MIC	15.62	250.00	31.25	31.25	15.62
		MFC	31.25	500.00	62.50	62.50	31.25
	Ethyl alcohol (70%)	MIC	62.50	NA	NA	62.50	7.80
		MFC	125.00	NA	NA	125.00	15.62
*C. annua* L.	Total crude extract	MIC	62.50	125.00	1.97	62.50	15.62
		MFC	125.00	250.00	3.90	125.00	31.50
	Ethyl alcohol (96%)	MIC	250.00	125.00	15.62	15.62	62.50
		MFC	500.00	250.00	31.25	62.50	125.00
	Ethyl alcohol (70%)	MIC	NA	125.00	7.80	125.00	15.62
		MFC	NA	250.00	15.62	250	15.62
Control	Benozed 25%	MIC	62.50	125.00	0.90	3.90	1.90
		MFC	125.00	250.00	1.90	7.90	3.90

NA: No activity.

#### Determination of ergosterol content in the plasma membrane

**Fig. 10:**
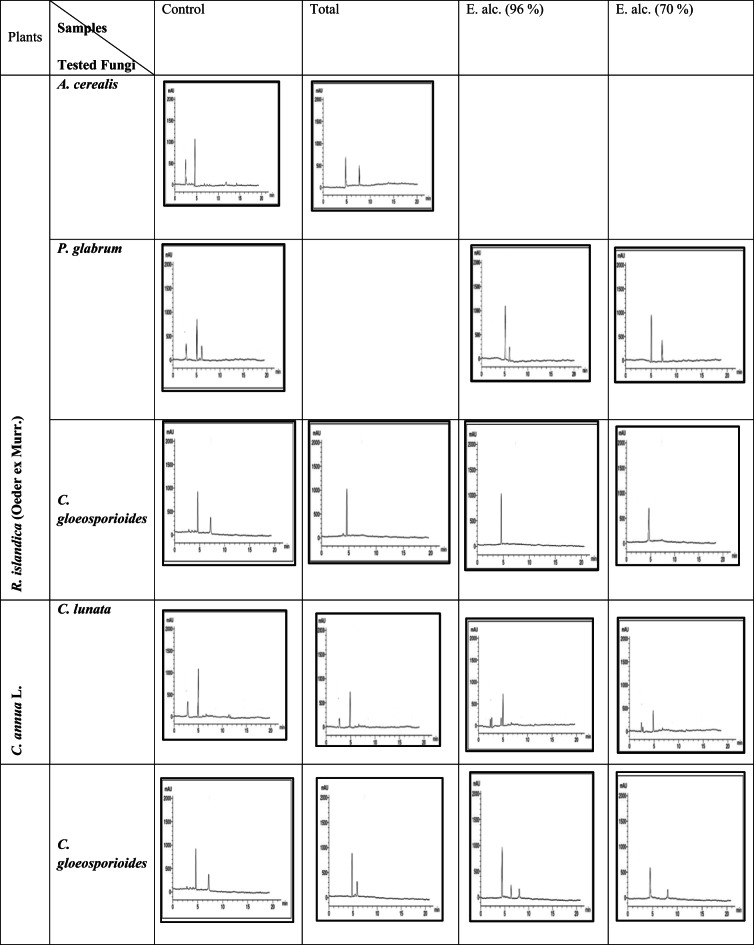
Charts of ergosterol content in tested fungal species and by total and ethyl alcohol (96% &70%) extracts of two plants under investigation.

Ergosterol is an essential functional component of the plasma membrane. The effect of sub- inhibitory concentration of ethyl alcohol (96% & 70%) extracts of the two plants under investigation on this vital content is showing in (Table 6 and Fig. 10). The results showed a significant reduction in ergosterol content in fungi treated with both plants. The strongest reduction was recorded in *C. lunata* and *C. gloeosporioides* treated with *C. annua* extract (49.93% and 47.7%, respectively). Similarly, *P. glabrum* and *C. gloeosporioides* treated with *R. islandica* extract exhibited marked reductions (47.2% and 42.58%, respectively).

These findings confirm that the antifungal effect of the extracts is associated with inhibition of ergosterol biosynthesis, leading to disruption of the fungal cell membrane.

**Table 6:** Reduction percent of ergosterol content in tested fungal species by total and Ethyl alcohol (96% &70%) extracts of two plants under investigation.

Plants	Samples Tested Fungi	Control	Total crude extract	*R*%	Ethyl alcohol (96%)	*R*%	Ethyl alcohol (70%)	*R*%
*R. islandica* (Oeder ex Murr.)	*A. cerealis*	17.00	10.40	38.80	NA	NA	NA	NA
	*P. glabrum*	19.51	NA	NA	11.90	39.00	10.30	47.20
	*C. gloeosporioides*	14.23	12.09	15	10.04	15.39	8.17	42.58
*C. annua* L.	*C. lunata*	22.15	18.10	18.28	14.17	36.00	11.09	49.93
	*C. gloeosporioides*	14.23	7.44	47.70	10.51	26.14	9.70	31.83

R%: Percent of reduction. NA: No Activity.

#### Observing morphological changes under AFM

**Fig. 11:**
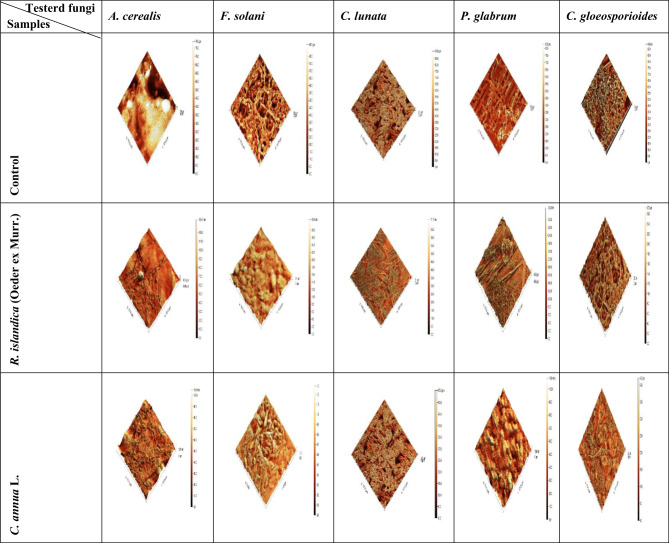
Topographic images of control and treated five tested fungi with total ethanol (70%) extracts of the two plants under investigation.

**Fig. 12:**
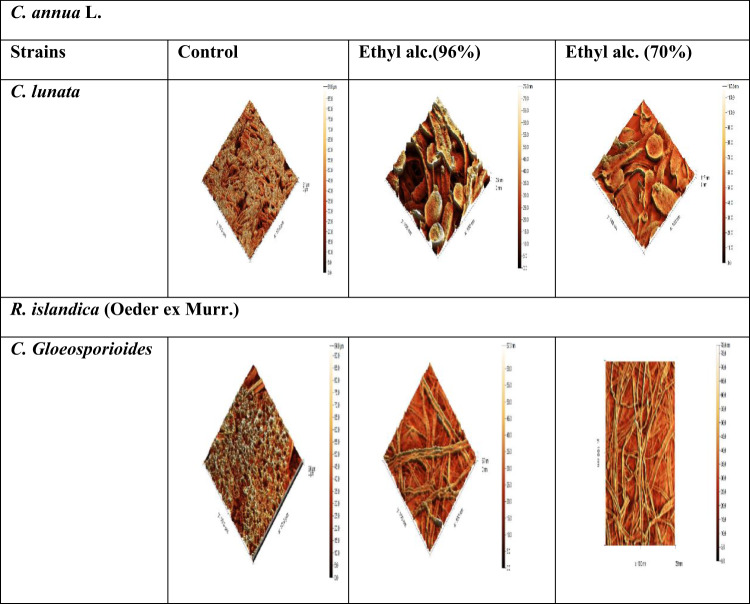
Topographic images of control and treated five tested fungi with successive extracts (ethyl alcohol 96%& ethyl alcohol 70%) of the two plants under investigation.

The changes in cell morphology induced by total and ethyl alcohol (96% &70%) extracts of two plants under investigation were examined using (AFM). AFM images give not only qualitative information of biological sample but also quantitative measurements at nanometer level. Therefore, it easy to make accurate comparison between treated and untreated samples. Since AFM is capable of providing a precise 3D map of the cell surface in the X, Y and Z dimentions on a sub micrometer scale, the Z axis (Z hight) value can be used to describe the effect of total extracts of two investigated plants against all tested fungi and ethyl alcohol (96% &70%) extracts against the most effected fungi. Variations in the Z hight of untreated and treated fungi with total extracts were apparent from the 3D images in (Fig. 11). The Z hights and roughness of untreated and treated *A. cerealis*,* F. Solani*,* C. lunata*,* P. Glabrum* and *C. Gloeosporioides* with total extract of *Rorippa islandica* (Oeder ex Murr.), *Carrichtera annua* L. appeared in (Table 7). As showed Z hights of untreated fungi (*A. cerealis*,* F. Solani*,* C. lunata*,* P. Glabrum* and *C. Gloeosporioides*) were found to be (79, 88, 91, 83 and 94) nm, respectively and after treatment with *R. islandica* (Oeder ex Murr.) were found to be (134.5, 30.9, 71.2, 330 and 27) nm, respectively and for *C. annua* L. (104.9, 1.3, 55.3, 108.4 and 42.1) nm, respectively. According to the showed result, it appeared that roughness of all tested treated fungi with the two total plant extracts under investigation were lower than roughness of untreated tested fungi except for *P. Glabrum* the roughness of it before treating (8.25 nm) was lower than roughness of it after treating with the two total plant extracts of *R. islandica* (Oeder ex Murr.) and *C. annua* L. (24.17 and 11.57) nm respectively. Also, Variations in the Z hight of untreated and treated fungi with the best of successive extracts (ethyl alc. 96% and ethyl alc. 70%) were apparent from the 3D images in (Fig. 12). The Z hights and roughness of untreated and treated *C.lunata* and *C. Gloeosporioides* with the best successive extracts (ethyl alc. 96% and ethyl alc. 70%) of *Rorippa islandica* (Oeder ex Murr.) and *Carrichtera annua* L. appeared in (Table 8). As showed Z hights of untreated fungi (*C. lunata* and *C. Gloeosporioides*) were found to be (91 and 94) nm respectively and after treatment of *C. Gloeosporioides* with *R. islandica* (Oeder ex Murr.) (ethyl alc. 96% and ethyl alc. 70%) were found to be (67and 78) nm respectively and after treatment of *C. lunata* with. *Carrichtera annua* L. (ethyl alc. 96% and ethyl alc. 70%) was found to be (75 and 117.5) nm, respectively.

**Table 7:** Morphological changes under (AFM) in tested fungal species by control and treated five tested fungi with total ethanol (70%) extracts of the two plants under investigation.

Fungal strains	Control		*Rorippa islandica *(Oeder ex Murr.)		*Carrichtera annua* L.	
	Z hight nm	Roughness	Z hight nm	Roughness	Z hight nm	Roughness
*A. Cerealis*	79.00	16.48	134.5	8.29	104.9	7.96
*F. Solani*	88.00	13.69	30.90	3.11	1.30	0.13
*C. lunata*	91.00	15.84	71.20	7.47	55.3	9.50
*P. Glabrum*	83.00	8.25	330.00	24.17	108.40	11.57
*C. Gloeosporioides*	94.00	16.52	27.00	3.94	42.10	4.50

**Table 8:** Morphological changes under (AFM) in tested fungal species by control and treated five tested fungi with successive extracts of the two plants under investigation.

Samples	Tested fungi									
	Control		*R. islandica* (Oeder ex Murr.)				*C. annua* L.			
			Ethyl alcohol (96%)		Ethyl alcohol (70%)		Ethyl alcohol (96%)		Ethyl alcohol (70%)	
	Z hight	Roughness	Z hight	Roughness	Z hight	Roughness	Z hight	Roughness	Z hight	Roughness
*C. lunata*	91.00	15.84	**-**	**-**	**-**	**-**	75.00	16.70	117.50	11.50
*C. Gloeosporioides*	94.00	16.52	67.00	6.80	78.00	8.70	**-**	**-**	**-**	**-**

## Discussion

Food security, defined as universal physical, social, and economic access to safe and nutritious food, is currently threatened by crop diseases and the consequences of intensive fungicide use. Although chemical fungicides are widely employed to control these diseases, they are associated with environmental damage, human exposure, and residue accumulation on crops. Frequent emergence of resistance further reduces their effectiveness, generating a strong demand for safer, alternative chemotherapeutic agents^32,33^. This has spurred active research into natural products, particularly plant extracts containing bioactive secondary metabolites such as alkaloids, quinones, flavonoids, glycosides, saponins, tannins, and terpenoids, which offer potential in controlling plant pahogens^33,34^. Historically, inappropriate pesticide application—including aerial spraying—has caused human toxicity, environmental contamination, and the evolution of resistant or more harmful species. Consequently, authorities emphasize the proper use of pesticides to protect human, animal, and environmental health^1^. Biopesticides, derived from natural sources or living organisms^4^, are increasingly adopted in environmentally friendly integrated crop management (ICM), aligning with global trends that reduce synthetic chemical usage.

In this study, environmentally friendly antifungal agents were evaluated. We used 70% ethanolic crude extracts and six successive extracts from two *Brassicaceae* plants, *Rorippa islandica* and *Carrichtera annua*, collected from Egypt’s Northwestern Coastal Region (Marsa Matrouh). These extracts were tested against five pathogenic fungi: *A. cerealis* MT808477, *F. solani* OK464437, *C. lunata* OM432028, *P. glabrum* Op694171, and *C. gloeosporioides* OP177948OP177948. Both plant extracts revealed potential antifungal activity and significant reduction in the ergosterol content.

GC-MS analysis revealed the presence of several bioactive compounds, including hydrocarbons, oxygenated molecules, fatty acids, fatty acid esters, sulfur-containing compounds, amides, alkaloids, sterols, and terpenes^35^. These results are consistent with the work of other researchers, such as the findings on ethanolic leaf extracts of *Hellenia speciosa* which contain a similar range of compounds, contributing to its diverse pharmacological properties, such as antioxidant, antimicrobial, anticancer, antidiabetic, and anti-inflammatory activities^36^. Different studies have reported that Fatty Acid Methyl Esters (FAMEs) derived from vegetable oils and plant seeds demonstrate significant antifungal activity, often surpassing the effectiveness of some conventional drugs. These compounds show low Minimum Inhibitory Concentration (MIC) values against a variety of pathogens, including *Paracoccidioides spp.*, *Candida* species, and *Aspergillus*. Notably, unsaturated esters like methyl linoleate are especially potent in their antifungal action^37,38^. The isolated sterols (e.g., stigmasterol, beta-sitosterol) and triterpenoid esters from plants and fungi exhibit moderate to significant antifungal activity^39^. Key components identified in this study, such as 13-docosenamide (Z) in *R. islandica*, is a fatty acid amide, exhibits broad-spectrum antifungal properties^40,41^, and inhibited *Valsa mali* (apple canker pathogen) by disrupting hyphal membranes and causing cellular content leakage^42^. GC-MS analysis showed that 9-Octadecenamide, also known as oleamide, was the second abundant compounds in chemical profile of *R. islandica* has been identified as a major component in several plant and microbial extracts with reported antimicrobial properties. In recent studies, extracts rich in 9-octadecenamide demonstrated inhibitory effects against phytopathogenic fungi^43,44^. Our GC-MS analysis of *R. islandica* extract identified β-sitosterol, a plant sterol structurally similar to cholesterol. The presence of this compound is significant, as previous studies have confirmed its antimicrobial properties. For instance, research demonstrated that the presence of β-sitosterol in *Azadirachta indica* leaf extracts was responsible for inhibiting fungal growth^45,46^. GC-MS analysis of both *Rorippa islandica* and *Carrichtera annua L.* extracts revealed the presence of hexadecanoic acid, also known as palmitic acid. This saturated fatty acid is widely found in plants, animals, and microorganisms, and its presence is significant because of its documented antimicrobial properties, hexadecanoic acid is a common compound in the leaves of *Pistia stratiotes* L. and *Eichhornia crassipes* (Mart.), with its derivatives such as E-11-hexadecenoic acid, ethyl ester exhibiting antifungal activity. This supports the notion that fatty acids play a crucial role in the antifungal mechanisms of these plant extracts^47^. Microbial degradation of n-hexadecanoic acid is initiated at the oxidized end of the molecule through a process called beta-oxidation. In contrast, the microbial oxidation of saturated hydrocarbons like n-hexadecane is more difficult to initiate because they lack this readily available oxidized site^48^. Higher plants use linolenic and linoleic acid as substrates to produce a variety of trihydroxy oxylipins with antifungal properties. For example, 9(S), 12(S), 13(S)-trihydroxy-10(E)-octadecenoic acid, produced in plants infected with the rice blast fungus *Magnaporthe grisea*, exhibits antifungal activity against the fungus^49^. Benzene derivatives such as benzothiazoles, phenol, and trimethylphenol effectively inhibit *Fusarium oxysporum* via combined suppression of mycelial growth and spore germination^50^.

Ethanol extracts and successive fractions exhibited strong antifungal activity, reflecting solvent-dependent extraction of secondary metabolites^51^. Gallic acid, which was detected as the main phenolic compound in both investigated plants by HPLC, inhibits the growth of crop-infecting fungi such as *Pythium aphanidermatum* and *Magnaporthe grisea*, with complete growth cessation at higher concentrations^52^ and strong antifungal activity against *Fusarium solani*^53^. Gallic acid’s activity contributed to antifungal mechanisms through induces apoptosis-like cell death, involving mitochondrial dysfunction and increased reactive oxygen species^54^ and bind to fungal carbonic anhydrase, potentially reducing virulence and drug resistance^55^. Our plants were rich by flavonoids which exhibit multifaceted antifungal activity through the fungal cell cycle, inhibit hyphal growth, and prevent biofilm formation, which is crucial for pathogenicity and drug resistance, especially in *Candida* species^56,57^. In another study, flavonoids can block fungal efflux pumps, reduce drug resistance and enhancing the efficacy of conventional antifungals^56,58^. Our results documented the presence of catechins in two plants under investigation, but it was with high concentration in *C. annua* extract. Catechin and its derivatives also show activity against *Aspergillus niger* and *Penicillium citrinum*^59^. Rutin, a plant-derived flavonoid, is observed only in *R. islandica* extract is often identified as a key polyphenol in medicinal plant extracts with antifungal properties, suggesting it may contribute to the overall activity of these extracts and like other polyphenols, rutin may damage fungal cell membranes or cell walls, contributing to its antifungal action^60^. In other study, flavonoid-rich extracts of *A. maurorum* Medic, including kaempferol and quercetin derivatives, impair microbial viability by interfering with DNA replication and cell division^61,62^,

Mechanistically, ergosterol in fungal membranes is a key target. Quantitative analysis indicated its depletion following exposure to plant extracts, suggesting compromised membrane integrity^63,64^. Some medicinal plant extracts, such as those from *Tulbaghia violacea*, inhibit enzymes in the ergosterol biosynthetic pathway, causing accumulation of upstream intermediates and a dose-dependent reduction in ergosterol production in *Aspergillus flavus*. This disruption weakens the fungal cell membrane, ultimately inhibiting fungal growth^65^. Terpenes have been demonstrated to change the permeability and fluidity of the lipid bilayer membrane by penetrating the fungal cell wall and gaining access between the fatty acid chains that comprise it. The cell wall may break down and adhere to host surfaces less well as a result of these alterations, in addition to other effects that include disruption of the plasma membrane, loss of cell content, cytoplasmic coagulation, and cell lysis^66^.

In the context of fungal pathogenesis, the cell surface plays a critical role in mediating host-pathogen interactions, including attachment to host tissues, immune evasion, and the progression of disease^67^. Similar to bacteria, the molecular interactions and surface composition of fungi are crucial for the spread of infection^68,69^. The properties of pathogen surfaces are determined by various macromolecules, such as (glyco) proteins, lectins, polysaccharides, and lipids^69–71^. A significant challenge lies in understanding the structural organization of these molecules at the fungal surface and how they interact with their environment.

Over the last decade, Atomic Force Microscopy (AFM) has emerged as an essential tool in nanobiotechnology to address these challenges. AFM’s ability to image biological cell surfaces under physiological conditions with nanoscale resolution and to probe cell surface properties with piconewton sensitivity makes it invaluable for this research^72,73^.

AFM topographic imaging in this study showed that the total ethanol extracts and the best successive ethanol fractions (96% and 70%) from *R. islandica* (Oeder ex Murr.) and *C. annua* caused significant morphological changes on the surfaces of several fungal species, including *A. cerealis*,* F. solani*,* C. lunata*,* P. glabrum*, and *C. gloeosporioides*. This altered morphology is likely due to the natural bioactive compounds in these extracts, which interfere with the architecture of the fungal morphology, including the cell membranes and functional groups on the surface^74^. Furthermore, our results are consistent with Behbehani et al.^75^, who reported that fungi can undergo serious morphological changes in response to plant extract treatment. These changes might represent an adaptive strategy by the fungi to mitigate the toxic effects of antifungal molecules, allowing them to survive in a hostile environment by masking the impact of the stress molecules.

## Conclusion

In conclusion, our study confirms the promising potential of *R. islandica* and *C. annua L.* extracts as natural fungicides. Their efficacy is linked to a complex mixture of bioactive compounds that act through multiple mechanisms, including membrane disruption and physical deformation of the cell wall. Future research should focus on isolating and characterizing the individual compounds responsible for the highest antifungal activity to develop more targeted and effective biopesticide formulations.

## Data Availability

Data is provided within the manuscript.
